# Genetic Susceptibility to Antisynthetase Syndrome Associated With Single-Nucleotide Variants in the *IL1B* Gene That Lead Variation in IL-1β Serum Levels

**DOI:** 10.3389/fmed.2020.547186

**Published:** 2020-11-24

**Authors:** Marco Antonio Ponce-Gallegos, Espiridión Ramos-Martínez, Adriana García-Carmona, Mayra Mejía, Karol J. Nava-Quiroz, Gloria Pérez-Rubio, Enrique Ambrocio-Ortiz, Montserrat I. González-Pérez, Ivette Buendía-Roldán, Jorge Rojas-Serrano, Ramcés Falfán-Valencia

**Affiliations:** ^1^HLA Laboratory, Instituto Nacional de Enfermedades Respiratorias Ismael Cosio Villegas, Mexico City, Mexico; ^2^Unidad de Medicina Experimental, Facultad de Medicina, Universidad Nacional Autónoma de México, Mexico City, Mexico; ^3^Interstitial Lung Disease and Rheumatology Unit, Instituto Nacional de Enfermedades Respiratorias Ismael Cosio Villegas, Mexico City, Mexico; ^4^Translational Research Laboratory on Aging and Pulmonary Fibrosis, Instituto Nacional de Enfermedades Respiratorias Ismael Cosio Villegas, Mexico City, Mexico

**Keywords:** antisynthetase syndrome, IL1B gene, IL1-beta, SNP, anti-Jo1, genetic association

## Abstract

The antisynthetase syndrome (ASSD) is an autoimmune disorder characterized by myositis, arthritis, mechanic's hands, fever, Raynaud phenomenon, and interstitial lung disease (ILD). We aimed to evaluate single-nucleotide polymorphisms in the *interleukin 1B* (*IL1B*) gene and their association between ILD with antisynthetase autoantibodies, as well as IL-1β serum levels. The most frequent antisynthetase autoantibody was anti-Jo1. The most frequent tomographic pattern was non-specific interstitial pneumonia, whereas in the anti-Jo1 subjects, it was organized pneumonia. Anti-Jo1 patients tend to have more significant arthritis, and Raynaud phenomenon have higher levels of creatinine phosphokinase. In the *IL1B* gene, the GG genotype and G allele of rs1143634 [odds ratio (OR) = 2.21 and OR = 2.60, respectively, *p* < 0.05] are associated with an increased risk, as well as with the dominant and recessive models (*p* < 0.05). This finding is maintained after logistic regression analysis adjusting for potential confounding variables (*p* < 0.05). Subjects with the rs16944/AG heterozygous genotype had higher serum levels of IL-1β compared to homozygous (*p* < 0.05). In conclusion, rs1143634 is associated with a higher risk of ASSD. Also, the GA genotype is associated with higher levels of IL-1β in ASSD patients.

## Introduction

The antisynthetase syndrome (ASSD) is an autoimmune disorder characterized by diverse clinical manifestations, including myositis, arthritis, mechanic's hands, fever, Raynaud phenomenon, and interstitial lung disease (ILD), as well as the presence of aminoacyl-transfer RNA synthase (ARS) autoantibodies (1, 2). The ASSD was firstly associated with idiopathic inflammatory myopathies (IIMs); however, previously, it has been described that many of these patients may have only slight myositis clinical manifestations and not fulfill with the Bohan and Peter criteria for an inflammatory myopathy (3–5).

The ARS autoantibodies include anti-Jo1 (anti-histidyl), anti-EJ (anti-glycyl), anti-OJ (anti-isoleucyl), anti-PL7 (anti-threonyl), anti-PL12 (anti-alanyl), anti-SC (anti-lysil), anti-KS (anti-asparaginyl), anti-JS (anti-glutaminyl), anti-Ha or anti-YRS (anti-threonyl), anti-tryptophanyl, and anti-Zo (anti-phenylalanyl), with anti-Jo1 being the most common (6). Differences have been described according to the different ARS autoantibodies. For example, PL7 and PL12 are associated with early and severe ILD (7, 8). Also, anti-Jo1 is associated with more muscle involvement and better prognosis (9, 10).

However, despite the clinical characteristics of ASSD have been widely described and are a topic of intensive research, little is known about this nosological entity's genetic background. Previous reports of single-nucleotide polymorphisms (SNPs) in proinflammatory genes associated with IIM, such as *PTPN22, PLCL1*, and *TNF*, have been described (11–13). However, despite that interleukin 1β (IL-1β) has been associated with other autoimmune disorders such as rheumatoid arthritis (RA) and systemic lupus erythematosus (SLE), the association between SNPs in the *IL1B* gene and IIM has been poorly described (14, 15). There is only one previous report of *IL1RN* VNTR polymorphism associated with dermatomyositis (DM) in a Bulgarian population (16). On the other hand, studies related to ILD with ARS autoantibodies are relatively scarce. We aimed to evaluate SNPs in *IL1B*, IL-1β serum levels, and their association between ILD with ARS autoantibodies.

## Materials and Methods

### Subjects Included

#### Case Group

One hundred fifty-four patients with ASSD diagnosis were included. All of them were evaluated and managed in the Interstitial Lung Disease and Rheumatology Unit (ILD&RU) at the Instituto Nacional de Enfermedades Respiratorias Ismael Cosio Villegas (INER) in México City. Patients in the ILD&RU are evaluated by a multidisciplinary group (pulmonologists, radiologists, and rheumatologists). Included patients were ≥18 years old, born as Mexican mestizos (MMs), no biological relation among themselves or with the patients or controls, with the diagnosis of ILD confirmed by high-resolution computed tomography (HRCT) and be positive to at least one of the following autoantibodies: anti-Jo1, anti-PL7, anti-PL12, and anti-Ej (subjects positive only for anti-OJ antibody were excluded). According to the manufacturer's instructions, all autoantibodies were measured by the Myositis Profile 3 immunoblot 16 strips EUROLINE panel (EUROIMMUN AG, Lübeck, Germany). Also, we included baseline pulmonary function tests, such as single-breath carbon monoxide diffusing capacity (DLco) and spirometry. Furthermore, baseline serum creatinine phosphokinase (CPK) levels were recorded, as well as the history of Raynaud phenomenon, arthritis, mechanic's hands, fever, and smoking history. Patients were evaluated between January 2008 and January 2019. Besides, the case group was divided into anti-Jo1 and non–anti-Jo1 patients for further analyses.

#### Control Group

A group of five hundred six healthy volunteer subjects (HS) was also included. These subjects had the following characteristics: clinically healthy (with neither chronic nor acute diseases self-reported), ≥18 years old, men and women, born as MMs (no biological relation among themselves or with the patients or controls), and no history of family pulmonary or inflammatory/autoimmune diseases. All subjects had at least three generations born in Mexico (parents and grandparents) and were considered MMs. We have previously demonstrated that this criterion is a good proxy of Mexican ancestry evaluated by ancestry-informative markers (17). All participants underwent a background questionnaire of inherited pathologies, excluding subjects who reported suffering from some lung and chronic inflammatory disease.

#### Ethics Approval and Informed Consent

The Institutional Committees for Research, Ethics in Research, and Biosecurity of the INER approved this study (approval code numbers: C08-17, B11-19). All participants were previously invited to participate in the protocol; they signed a written informed consent document and provided with a privacy statement describing personal data protection.

All experiments were performed following the relevant guidelines and regulations. The STREGA (STrengthening the REporting of Genetic Association) guidelines were considered to design this genetic association study (18).

#### DNA Extraction

The DNA was extracted from peripheral blood cells via venipuncture using EDTA tubes from all 668 subjects' using the commercial BDtract Genomic DNA isolation kit (Maxim Biotech, San Francisco, CA, USA). The DNA was quantified by UV absorption spectrophotometry at the 260-nm wavelength employing a NanoDrop 2000 device (Thermo Scientific, Wilmington, DE, USA). Contamination with organic compounds and proteins was determined by measuring the ratio absorbance at 280 and 260 nm. Samples were considered of good quality when the ratio was ~1.8.

#### SNP Selection

SNPs were selected based on a bibliographic search in PubMed (NCBI), identifying polymorphisms in *IL1B* previously associated with IIM and other autoimmune diseases, such as RA and SLE. Additionally, according to the HapMap project, we considered a minor allelic frequency (MAF) higher than 5% in the Mexican population in Los Angeles. Table 1 summarizes the principal characteristics of the evaluated SNPs.

**Table 1:** Characteristics of SNPs evaluated.

**SNP**	**Position (pb)**	***HWE***	***p*-value**	**MAF**	**Alleles**
rs1143634	112832813	2.9855 × 10^13^	0.0012	0.09	G:A
rs16944	112837290	0.3876	0.3136	0.36	A:G
rs1143623	112838252	0.6825	0.0893	0.4	G:C

*HWE, Hardy–Weinberg equilibrium; MAF, minor allele frequency; SNP, single-nucleotide polymorphism*.

#### SNP Genotyping

The SNPs' allele discrimination was performed using commercial TaqMan probes (Applied Biosystems, San Francisco, CA, USA) at a concentration of 20 × in total subjects included. SNPs evaluated were rs1143634, rs16944, and rs1143623 in the *IL1B* gene, using quantitative polymerase chain reaction (qPCR) in a 7300 Real-Time PCR System (Applied Biosystems/Thermo Fisher Scientific Inc., Singapore), and the analysis performed by sequence detection software version 1.4 software (Applied Biosystems, CA, USA). Also, three controls without template (contamination controls) included each genotyping plate, and 5% of the genotyped in duplicate as controls for allele assignment.

Besides, to determine the haplotype structure in the *IL1B* gene associated with ASSD susceptibility, we applied Haploview software version 4.2.

#### Measurement of IL-1β Serum Levels

Once performing the association analyses, we selected sera samples from 62 ASSD patients, carrying genotypes of the three SNPs evaluated. Sample selection was performed to identify representability for each genotype from all SNPs. For the rs1143634, we selected 58 with GG and 4 with GA genotypes. For the rs16944 group, we included 33 with AA, 25 with AG, and 4 with GG genotypes. For the rs1143623, we chose 5 with CC, 28 with CG, and 29 with GG genotypes. Differences in the patients' number, according to genotypes, are due to serum samples availability and the genotype frequencies. The serum levels were measured by the enzyme-linked immunosorbent assay (ELISA) technique; the detectable range of IL-1β was 14.06–900 pg/mL (human IL-1 beta ELISA Kit, ab214025, Abcam Plc, Cambridge, UK). A total of 50 μL of serum for each sample was centrifuged and prepared for analysis, following the manufacturer's protocol.

#### Statistical Analysis

The differences between groups were assessed by determining and comparing the allele and genotype frequencies. The statistical significance was assessed using SPSS v20.0 (SPSS Inc., Chicago, IL, USA) and Epi Info 7.1.4.0 statistical software (19). The allele and genotype frequencies between groups were analyzed using the χ^2^-test. The results were considered to be significant when *p* < 0.05; similarly, the odds ratios (ORs) with 95% confidence intervals (CIs) were estimated to determine the strength of the association. Comparisons made between ASSD and HS are shown. Also, the ASSD patients are divided into anti-Jo1+ and non–anti-Jo1.

A logistic regression analysis was performed to adjust for potential confounding variables [sex, age, and body mass index (BMI)] using Plink v. 1.07.

## Results

### Demographic Variables in Case and Control Groups

One hundred fifty-four patients with at least one ARS autoantibody and ILD diagnosed by HRCT were included, and 506 healthy subjects as the control group. The ASSD group was older and predominantly women compared with the HS group (*p* < 0.05). There were no significant differences in BMI.

Furthermore, 37% of the ASSD patients are smokers with 21 years of smoking, 5 cigarettes per day, and 6 pack-years' history. The most frequent clinical manifestation was arthritis (70.13%), followed by mechanic's hands, fever, and Raynaud phenomenon. Also, the most frequent ARS autoantibody was anti-Jo1 (43.51%), and the most frequent HRCT pattern was non-specific interstitial pneumonia (NSIP) (45.70%). Table 2 shows the complete results.

**Table 2:** Demographic and clinical variables from ASSD and HS groups and among ASSD anti-Jo1 and non–anti-Jo1.

**Variables**	**ASSD**	**HS**	***p*-value**	**Anti-Jo1**	**Non–anti-Jo1**	***p*-value**
	**(*n* = 154)**	**(*n* = 506)**		**(*n* = 67)**	**(*n* = 87)**	
**Age (years)**	57 (27–83)	45 (26–81)	<0.001	54 (41–73)	57 (38–75)	0.013
**Sex, female (%)**	109 (70.78)	216 (42.5)	<0.001	47 (70.15)	62 (71.26)	0.880
**BMI**	26.9 (13.3–45.5)	27.51 (15.82–52.03)	0.954	27.3 (23–35.9)	26.9 (15.6–39.4)	0.804
**Smoking status**						
Smoker, yes (%)	57 (37.01)			22 (32.84)	35 (40.23)	0.346
Years of smoking	21 (1–63)			18 (1–45)	23 (2–63)	0.431
Cigarettes per day	5 (1–40)			10 (1–40)	5 (1–20)	0.310
Tobacco index	6 (0.5–56)			7.1 (0.5–56)	4.6 (0.35–44)	0.446
**Pulmonary function**						
FVC % pre-bd	59 (32–114)			57 (32–114)	59 (33–109)	0.199
DLco	47 (10–110)			48 (10–102)	47 (12–110)	0.342
**Clinical manifestations**						
Arthritis	108 (70.13)			52 (77.61)	56 (64.37)	0.075
Mechanic's hands	82 (53.25)			40 (59.70)	42 (48.28)	0.159
Fever	76 (49.35)			37 (55.22)	39 (44.83)	0.201
Raynaud phenomenon	69 (44.81)			35 (52.24)	34 (39.08)	0.106
**CPK**	81 (18–7,210)			196.5 (24–7,210)	71 (18–5,619)	0.001
**Autoantibodies**						
Anti-Jo1 (%)	67 (43.51)					
Anti-PL12 (%)	55 (35.71)					
Anti-PL7 (%)	35 (22.73)					
Anti-EJ (%)	19 (12.34)					
**TAC**	*n* = 140			*n* = 61	*n* = 79	
NSIP (%)	64 (45.70)			21 (34.43)	43 (54.43)	0.018
OP (%)	41 (29.29)			26 (42.62)	15 (18.98)	0.002
UIP (%)	23 (16.43)			6 (9.84)	17 (21.52)	0.064
LIP (%)	6 (4.29)			3 (4.92)	3 (3.80)	0.745
No class (%)	4 (2.86)			3 (4.92)	1 (1.27)	0.414
Br-ILD (%)	2 (1.43)			2 (3.28)	0	N/A

*ASSD, antisynthetase syndrome; HS, healthy subjects; BMI, body mass index; CPK, creatine phosphokinase; DLco, single-breath carbon monoxide diffusing capacity; FVC, forced vital capacity; ILD, interstitial lung disease; LIP, lymphoid interstitial pneumonia; Br-ILD, bronchiolitis-associated interstitial lung disease. NSIP, non-specific interstitial pneumonia; OP, organized pneumonia; UIP, usual interstitial pneumonia. All values are expressed as median and minimum-maximum values. We used the Mann–Whitney U-test and Fisher exact test to make comparisons between groups. p < 0.05 was considered as significant*.

### Demographic Variables in Anti-Jo1 and Non–Anti-Jo1 Groups

Besides, we divided the case group into anti-Jo1 and non–anti-Jo1, comparing them to each other. Sixty-seven patients were included in the anti-Jo1 group, whereas 87 were included in the non–anti-Jo1 group. Patients included in the non–anti-Jo1 group were older than the anti-Jo1 subjects (*p* < 0.05). We did not find statistically significant differences between groups comparing sex, BMI, smoking status, pulmonary function (FVC-Pb and DLco), and clinical manifestations, such as mechanic's hands and fever and Raynaud phenomenon (*p* > 0.05). However, those subjects in the anti-Jo1 group tended to present more arthritis (*p* = 0.075). Also, anti-Jo1 patients present the most significant muscle involvement, represented by higher levels of CPK (*p* = 0.001). Interestingly, the most frequent HRCT pattern in the anti-Jo1 group was organized pneumonia (OP) (42.62 vs. 18.98%, *p* = 0.002), whereas in the non–anti-Jo1 group, it was NSIP (54.43 vs. 34.43%, *p* = 0.018). Complete results are shown in Table 2.

### Allele and Genotype Frequencies

We evaluated three SNPs (rs1143623, 1143634, and rs16944) in the *IL1B*. Table 3 shows the full genetic association results.

**Table 3:** Allele and genotype frequencies and genetic models of *IL1B* SNPs in case and control groups.

**Model**	**ASSD**		**HS**		***p*-value**	***p*-value Bonferroni correction**	**OR**	**(95% CI)**
	***n* = 154**	***F* (%)**	***n* = 496**	***F* (%)**				
**rs1143634**								
**Genotypes**								
GG	142	92.21	418	84.27	0.013	0.039	2.21	1.17–4.17
GA	11	7.14	54	10.89	0.18	NA	0.63	0.32–1.24
AA	1	0.65	24	4.84	0.015	0.045	0.13	0.02–0.96
**Alleles**								
G	295	95.78	890	89.72	0.001	0.003	2.60	1.44–4.70
A	13	4.22	102	10.28			0.38	0.21–0.70
**Dominant**								
GG	142	92.21	418	84.27	0.013	0.039	2.21	1.17–4.17
AG + AA	12	7.79	78	15.73			0.45	0.24–0.86
**Recessive**								
GG + GA	153	99.35	472	95.16	0.015	0.045	7.78	1.04–57.99
AA	1	0.65	24	4.84			0.13	0.02–0.96
**rs16944**								
**Genotypes**			*n* = 506					
AA	69	44.81	197	38.93	0.19	NA	1.27	0.88–1.83
AG	68	44.16	248	49.01	0.29		0.82	0.57–1.18
GG	17	11.04	61	12.06	0.73		0.91	0.51–1.60
**Alleles**								
A	206	66.88	642	63.44	0.27	NA	1.16	0.89–1.52
G	102	33.12	370	36.56			0.86	0.66–1.13
**Dominant**								
AA	69	44.81	197	38.93	0.19	NA	1.27	0.88–1.83
GA + GG	85	55.19	309	61.07			0.79	0.55–1.13
**Recessive**								
AA + GA	137	88.96	445	87.94	0.73	NA	1.10	0.62–1.95
GG	17	11.04	61	12.06			0.91	0.51–1.60
**rs1143623**								
**Genotypes**			*n* = 501					
GG	62	40.26	171	34.13	0.16	NA	1.30	0.90–1.89
CG	73	47.40	246	49.1	0.71		0.93	0.65–1.34
CC	19	12.34	84	16.77	0.19		0.70	0.41–1.19
**Alleles**								
G	197	63.96	588	58.68	0.10	NA	1.25	0.96–1.63
C	111	36.04	414	41.32			0.84	0.61–1.04
**Dominant**								
GG	62	40.26	171	34.13	0.16	NA	1.30	0.90–1.89
CG + CC	92	59.74	330	65.87			0.77	0.53–1.11
**Recessive**								
GG + CG	135	87.66	417	83.23	0.19	NA	1.43	0.84–2.44
CC	19	12.34	84	16.77			0.70	0.41–1.19

*ASSD, antisynthetase syndrome; HS, healthy subjects. NA, not applied. p < 0.05 was considered as significant and was corrected by the Bonferroni test*.

### Case and Control Groups

In the HS group, we genotyped 496 HS for rs1143634, 506 for rs16944, and 501 for rs1143623. We did not find statistically significant differences with allele and genotype frequencies between case and control group comparison for the rs1143623 and rs16944, and neither with dominant nor recessive models (*p* > 0.05). However, for the rs1143634, we found significant association with an increased risk of ASSD with G allele (*p* = 0.001, OR = 2.60, 95% CI = 1.44–4.70), which are maintained after Bonferroni correction (*p* = 0.003); in contrast, the A allele offered a reduced risk (*p* = 0.001, OR = 0.38, 95% CI = 0.21–0.70) for ASSD.

Regarding genotype frequencies, we found a statistically significant association (*p* = 0.015) with a reduced risk of ASSD with AA genotype (OR = 0.13, 95% CI = 0.02–0.96). Conversely, we found a significant association (*p* = 0.013) with a higher risk of ASSD with GG genotype (OR = 2.21, 95% CI = 1.17–4.17). In addition, in the dominant model, we found significant association (*p* = 0.013) with a reduced risk with AG + AA genotypes (OR = 0.45, 95% CI 0.24–0.70) and higher risk with GG genotype (OR = 2.21, 95% CI = 1.17–4.17). Additionally, in the recessive model, we also found a significant association (*p* = 0.015) with reduced risk with AA genotype (OR = 0.13, 95% CI = 0.02–0.96) and higher risk with GG + GA genotypes (OR = 7.78, 95% CI = 1.04–57.99), these associations are maintained after Bonferroni correction (*p* = 0.045). The complete results' list can be consulted in detail in Table 3.

### Logistic Regression Analysis

Logistic regression was performed to adjust for possible confounding covariables. For rs1143634/A allele, we found statistically significant differences comparing ASSD vs. HS in additive model, suggesting a reduced risk of ASSD (*p* = 0.027, OR = 0.46, 95% CI = 0.23–0.92) and adjusting for sex (*p* = 1.21E-07) and age (*p* = 1.79E-14). Additionally, we found significant differences in adjusting for sex and age for the same comparison with rs16944/G and rs1143623/C (*p* < 0.05). The results are shown in Supplementary Table 1.

### Anti-Jo1+ and Non–Anti-Jo1 Groups

Regarding allele frequencies, we did not find statistically significant differences between the three SNPs evaluated. Besides, we did not find significant association when we compared genotype frequencies and neither with dominant and recessive genetic association models (*p* > 0.05). Complete results are shown in Table 4.

**Table 4:** Allele and genotype frequencies and genetic models of *IL1B* SNPs among the case group.

**Model**	**Anti-Jo1**		**Non–anti-Jo1**		***p*-value**	**OR**	**95% CI**
	***n* = 67**	***F* (%)**	***n* = 87**	***F* (%)**			
**rs1143634**							
**Genotypes**							
GG	64	95.52	78	89.66	0.23	2.46	0.64–9.47
GA	3	4.48	8	9.20	0.35	0.46	0.12–1.82
AA	0	0	1	1.15	NA		
**Alleles**							
G	131	97.76	164	94.25	0.16	2.66	0.71–9.87
A	3	2.24	10	5.75		0.38	0.10–1.39
**Dominant**							
GG	64	95.52	78	89.66	0.23	2.46	0.64–9.47
GA + AA	3	4.48	9	10.34		0.41	0.11–1.56
**Recessive**							
GG + GA	67	100	86	98.85	NA		
AA	0	0	1	1.15			
**rs16944**							
**Genotypes**							
AA	32	47.76	37	42.53	0.52	1.24	0.65–2.34
AG	28	41.79	40	45.98	0.60	0.84	0.44–1.60
GG	7	10.45	10	11.49	0.84	0.90	0.32–2.50
**Alleles**							
A	92	68.66	114	65.52	0.56	1.15	0.71–1.86
G	42	31.34	60	34.48		0.87	0.54–1.40
**Dominant**							
AA	32	47.76	37	42.53	0.52	1.24	0.65–2.34
AG + GG	35	52.24	50	57.47		0.81	0.43–1.54
**Recessive**							
AA + AG	60	89.55	77	89	0.84	1.11	0.40–3.10
GG	7	10.45	10	11		0.90	0.32–2.50
**rs1143623**							
**Genotypes**							
GG	27	40.30	35	40.23	0.99	1.02	0.63–1.63
GC	32	47.76	41	47.13	0.94	1.03	0.54–1.94
CC	8	11.94	11	12.64	0.90	0.94	0.35–2.48
**Alleles**							
G	86	64.18	111	63.79	0.94	1.02	0.63–1.63
C	48	35.82	63	36.21		0.98	0.62–1.57
**Dominant**							
GG	27	40.30	35	40.23	0.99	1	0.52–1.92
GC + CC	40	59.70	52	59.77		1	0.52–1.91
**Recessive**							
GG + GC	59	88.06	76	87.36	0.90	1.07	0.40–2.82
CC	8	11.94	11	12.64		0.94	0.35–2.48

*NA, not applied; SNP, single-nucleotide polymorphism*.

### Haplotype Analysis

**Figure 1:**
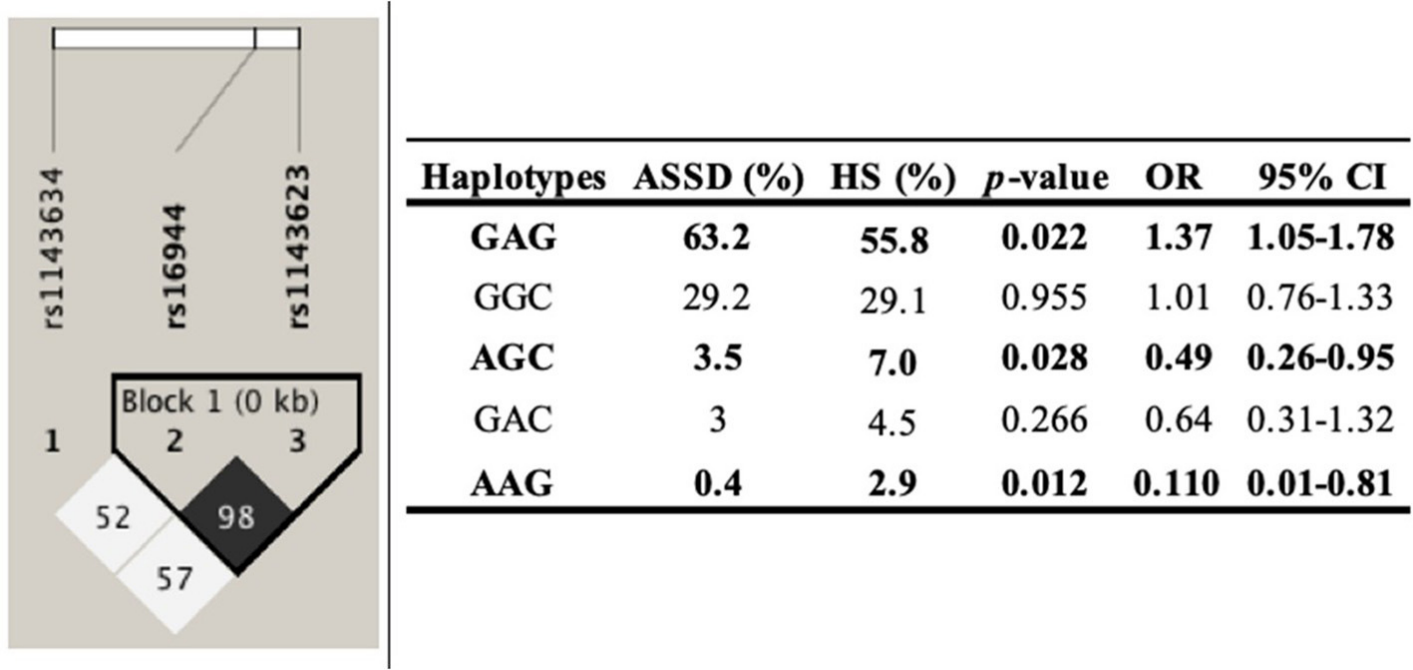
Haplotype analysis. ASSD, antisynthetase syndrome; HS, healthy subjects.

The haplotype analysis was carried out to determine its association with ASSD susceptibility. The analysis included the three SNPs in the *IL1B*, comparing ASSD vs. subjects in the control group. One of these polymorphisms evaluated (rs1143634) did not meet the Hardy–Weinberg equilibrium (*p* < 0.05). Haplotypes and their frequencies are summarized in Figure 1. The block shows that the haplotype shaped by rs16944 and rs1143623 is in high linkage disequilibrium (LD, *r*^2^ = 98). Moreover, according to the frequencies, GAG (conformed by all common alleles) haplotype is associated with a higher risk of ASSD (*p* = 0.022, OR = 1.37, 95% CI = 1.05–1.75). On the other hand, AGC (conformed by all minor alleles) and AAG (conformed by one minor allele and two common alleles) haplotypes are associated with reduced risk of ASSD (*p* = 0.028, OR = 0.49, 95% CI = 0.26–0.95; *p* = 0.012, OR = 0.110, 95% CI = 0.01–0.81).

### IL-1β Serum Levels

**Figure 2:**
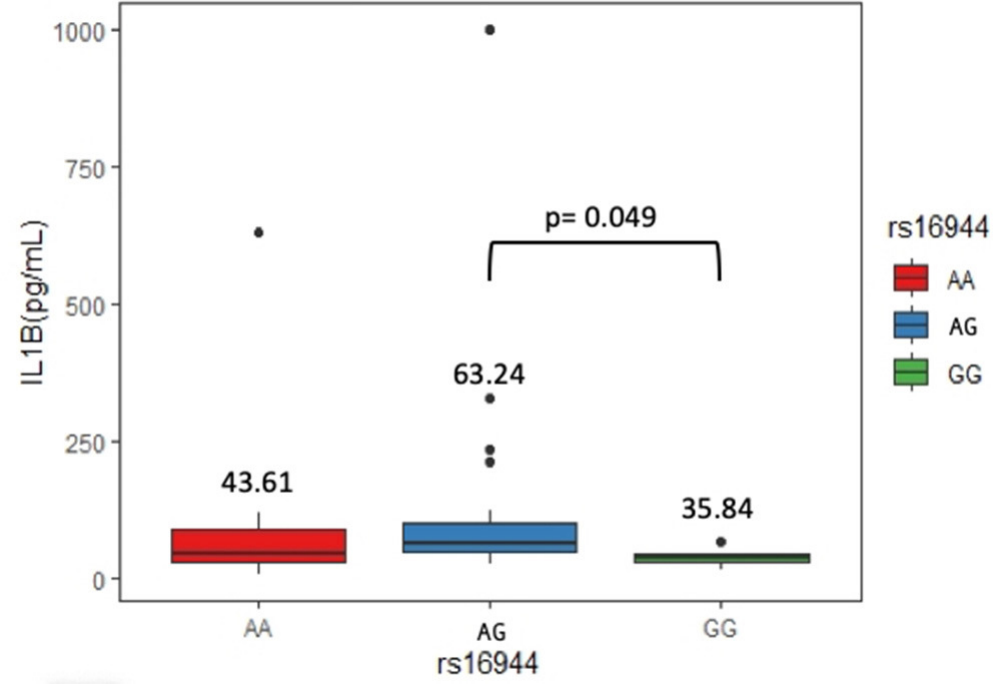
IL-1β serum levels between genotypes of rs61944 in the ASSD group.

Sixty-two serum samples from the ASSD group were selected, carrying genotypes of the three SNPs evaluated. We did not find significant differences between serum levels of IL-1β in rs1143623 and rs1143634. On the other hand, AG genotype (63.24 pg/mL) from rs16944 showed higher levels of IL-1β than homozygous genotypes [AA (43.61 pg/mL) and GG (35.84 pg/mL)], being statistically significant (*p* = 0.049). Figure 2 shows these results.

## Discussion

The ASSD is a rare systemic connective tissue disease, which usually affects joints, skin, muscles, and lungs. The clinical characteristics of the ASSD group showed that the median age was 57 years, and 70.6% were female. Also, arthritis was the most frequent clinical manifestation, whereas the most frequent ARS autoantibody was anti-Jo1 (41.36%). Also, NSIP was the most frequent tomographic pattern. These findings agree with two previous reports of our research group (1, 5).

Clinical manifestations can differ according to the different ARS autoantibodies. Pinal-Fernández et al. (10) showed that those anti-Jo1+ patients have more arthritis, muscle weakness, and Raynaud phenomenon. Rojas-Serrano et al. (6) described that anti-Jo1 patients had more arthritis, proximal muscle weakness, and higher levels of CPK than those non–anti-Jo1. These findings are similar to our current report and supported by the results shown in the largest ASSD patients cohort confirmed by the American and European NEtwork of Antisynthetase Syndrome collaborative group (20). They included 828 patients from 10 countries and 63 hospitals and found that anti-Jo1 was the most frequent ARS autoantibody, and these subgroups of patients had more muscle and articular involvement. These findings suggest that anti-Jo1 ARS could be associated with multiorgan involvement, whereas non–anti-Jo1 ARSs are mainly lung limited.

Interestingly, the most frequent tomographic pattern in anti-Jo1 patients was OP, whereas in non–anti-Jo1 patients, it was NSIP. These two tomographic patterns have been widely described as the most prevalent in the ASSD (6, 21), with inconsistent frequencies among studied populations. For example, in a previous study of our research group, we reported that in our ASSD cohort, the most frequent HRCT pattern was OP, followed by NSIP (1). Conversely, other studies have shown that NSIP is the most frequent tomographic pattern (7, 9, 22). This finding could be due to differences in the sample size, different inclusion criteria, and distinct population characteristics.

For most of the rheumatic diseases, a genetic susceptibility component has been established. SNPs located in proinflammatory gene cytokines have been associated with an important number of these autoimmune diseases. One of the most studied genes is *IL1B*, which has been associated in previous studies with RA and SLE (23–25). However, only a few studies are trying to find genetic associations between single-nucleotide variations and ASSD, with controversial results. Our analysis found some associations with rs1143634, being the first time that these associations in ASSD patients in an MM population are described. However, in a previous study by Beretta et al. (26) described that rs1143634 is associated with a more restrictive ILD pattern in patients with systemic sclerosis (SSc) in an Italian population. Although we found an association with a reduced risk of ASSD with the minor allele of rs1143634, this finding could suggest an indirect association with genetic variants that were not considered in this study, as Balding says in his report for candidate polymorphisms (27).

In contrast, Sugiura et al. (28), in a Japanese population, described that *STAT4* rs7574865 was associated with a higher risk of DM/polymyositis (PM) but not with the presence of ILD in this group of patients. This finding is shared with SSc, whereas STAT4 rs7574865 is associated with a higher risk of SSc and is also associated with reduced risk of SSc-related ILD in a Caucasian population (29).

Also, in a Chinese Han population, Chen et al. (30, 31) found two SNPs in *ETS1* (rs7117932 and rs6590330) and one SNP in *CCL21* (rs951005) associated with a higher risk of DM/PM and with ILD related to IIM. Conversely, the same research group found a decreased frequency of minor allele rs7731626-A (*ANKRD55*) in DM-ILD and DM/PM-ILD patients, suggesting that the A variant may be protective against DM/PM-ILD (32). Even though we did not evaluate the same SNP, this finding agrees with our results because we also found that rs1143634 minor allele could play a protective role in ASSD.

*IL1B* polymorphisms have also been evaluated in other ILDs because of its profibrotic activity inducing fibroblast proliferation via platelet-derived growth factor (33). Volobaev et al. (34) showed in their study that an SNP in *IL1B* (rs16944) was associated with a higher risk of anthracosilicosis in coal miners in Russia. Also, it has been described that WNT/β-catenin signaling induces IL-1β expression by alveolar epithelial cells in a murine model of pulmonary fibrosis and an up-regulation of IL-1β in bronchoalveolar lavage fluid (BALF) in bleomycin-induced lung fibrosis *in vivo* (35). These findings suggest that IL-1β could play an essential role in several ILDs because of its proinflammatory and profibrotic properties.

In our knowledge, this is the first time that ASSD patients are divided into two subgroups (anti-Jo1 and non–anti-Jo1) and compared, taking into consideration the different autoantibodies to make allele and genotype associations from SNPs. These analyses were conducted because of the clinical and prognostic differences between the autoantibodies spectrum reported by different cohorts (6, 8, 20).

To the best of our knowledge, we described for the first time three haplotypes associated with higher and reduced risk of ASSD, even in IIM. However, haplotypes in the *IL1B* gene have previously been described in other inflammatory diseases such as osteoarthritis, RA, and SLE (15, 36, 37). Although one of the three SNPs does not meet the Hardy–Weinberg equilibrium, it has been previously described that for mestizo populations, this criterion is not always necessary to establish genetic associations because of the recombination events. The MM population has been previously described as a rich genetic variability product of many years of genetic recombinations between ancestral populations (Amerindian), Caucasian, and African descendants (38, 39).

Our study found that the AG genotype of rs16944 is associated with higher levels of IL-1β in ASSD patients. Several studies have identified higher levels of IL-1β in patients with diverse rheumatic and ILDs. For example, serum levels of IL-1β in idiopathic pulmonary fibrosis patients were significantly increased compared to healthy controls, as well as in BALF (40). Also, a higher expression of IL-1α, IL-1β, and transforming growth factor β in muscle tissue has been previously reported in patients with IIM (41). Previous reports have established that genetic variants in the *IL1B* gene may contribute to differences in the expression, translation, and secretion of IL-1β. Hall et al. (42) demonstrated that rs16944 promoter polymorphism could induce a greater affinity of the transcriptional factors, promoting a higher expression of IL-1β, which concurs with our results. Iglesias-Molli et al. (43) showed that the rs16944 heterozygous genotype is associated with changes in mRNA expression of IL-1β in patients with type 2 diabetes, and this is in agreement with previous publications where an IL-1β haplotype in its promoter region was associated with increased IL-1β mRNA (44).

This study is not free of limitations. First, we evaluated only three SNPs in the *IL1B* gene, and one of them did not meet the Hardy–Weinberg equilibrium. Second, we did not include a control group of subjects with IIM without ILD because our center attends only to those with pulmonary involvement. Additionally, we only were able to measure the IL-1β in only a single part of our cohort. However, we have one of the largest sample sizes for genetic association in ASSD. Also, we made an intracase analysis to evaluate the relationship between the SNPs and the different ARS autoantibodies.

In conclusion, rs1143634/G is associated with a higher risk of ASSD. Also, rs61944/GG genotype has a higher frequency in those patients positive for the anti-Jo1 autoantibody, and the GA genotype is associated with higher levels of IL-1β in ASSD patients. Three haplotypes are associated with ASSD; two of them (AGC and AAG) are associated with reduced risk of ASSD, whereas one is associated with higher risk. More studies are required to elucidate the role of proinflammatory cytokines in the disease and to offer new therapeutic targets for these patients.

## Data Availability Statement

The datasets generated for this study can be found in online repositories. The names of the repository/repositories and accession number(s) can be found at: ClinVar system, VCV000869137, VCV000869138, and VCV000869139; https://www.ncbi.nlm.nih.gov/clinvar/?term=%22HLA%20Laboratory%2C%20Instituto%20Nacional%20de%20Enfermedades%20Respiratorias%20Ismael%20Cosio%20Villegas%22[submitter]+AND+%22IL1B%22[gene].

## Ethics Statement

The studies involving human participants were reviewed and approved by the Institutional Committees for Research, Ethics in Research, and Biosecurity of the Instituto Nacional de Enfermedades Respiratorias Ismael Cosio Villegas (INER) approved this study (approval code numbers: C08-17, B11-19). The patients/participants provided their written informed consent to participate in this study.

## Author Contributions

ER-M, JR-S, and RF-V: conceptualization. KN-Q and MG-P: data curation. MP-G and AG-C: formal analysis. IB-R: funding acquisition. ER-M, AG-C, KN-Q, EA-O, and MG-P: investigation. ER-M, AG-C, KN-Q, and EA-O: methodology. JR-S: project administration. MM, MG-P, IB-R, and JR-S: resources. MM, EA-O, and IB-R: software. MM, GP-R, IB-R, JR-S, and RF-V: supervision. MM and GP-R: validation. MM, GP-R, and JR-S: visualization. MP-G and RF-V: writing—original draft and writing—review and editing. All authors contributed to the article and approved the submitted version.

## Conflict of Interest

The authors declare that the research was conducted in the absence of any commercial or financial relationships that could be construed as a potential conflict of interest.
