# Supplementary material for: Genetic Susceptibility to Antisynthetase Syndrome Associated With Single-Nucleotide Variants in the IL1B Gene That Lead Variation in IL-1β Serum Levels
Source: Front Med (Lausanne). 2020 Nov 24;7:547186. doi: 10.3389/fmed.2020.547186 (PMC7732678; doi:10.3389/fmed.2020.547186)

**Supplementary Table 1.** Logistic regression analysis by co-variables in ASSD *vs.* HS

| **CHR** | **SNP** | **BP** | **A1** | **TEST** | **OR** | **95% CI** | ***P*-VALUE** |
| --- | --- | --- | --- | --- | --- | --- | --- |
| 2 | rs1143634 | 112832813 | 1 | ADD | 0.4635 | 0.234-0.918 | 0.02744 |
|  |  |  |  | SEX | 0.2983 | 0.191-0.467 | 1.21E-07 |
|  |  |  |  | AGE | 1.085 | 1.062-1.107 | 1.79E-14 |
|  |  |  |  | BMI | 1.032 | 0.985-1.081 | 0.1817 |
|  | rs16944 | 112837290 | 3 | ADD | 0.8356 | 0.602-1.159 | 0.2822 |
|  |  |  |  | SEX | 0.3013 | 0.193-0.470 | 1.19E-07 |
|  |  |  |  | AGE | 1.089 | 1.066-1.111 | 9.41E-16 |
|  |  |  |  | BMI | 1.033 | 0.987-1.082 | 0.1658 |
|  | rs1143623 | 112838252 | 2 | ADD | 0.7554 | 0.549-1.04 | 0.0858 |
|  |  |  |  | SEX | 0.3011 | 0.193-0.470 | 1.28E-07 |
|  |  |  |  | AGE | 1.088 | 1.066-1.111 | 1.05E-15 |
|  |  |  |  | BMI | 1.034 | 0.987-1.083 | 0.1553 |

Abbreviations and keys: ASSD: Antisynthetase syndrome; HS: Healthy subjects; CHR: Chromosome; SNP: Single nucleotide polymorphism; BP: Base pair location; A1: minor allele for each SNP; BMI: body mass index; OR: Odds ratio; 95% CI: 95% Confidence interval; ADD: Additive effect model.

**Supplementary Figure 1.** IL-1β serum levels between genotypes of rs1143634 and rs1143623 in the ASSD group


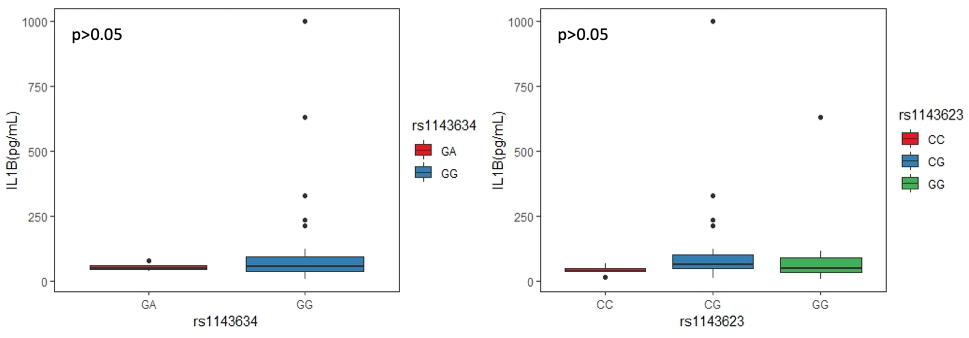

Supplement: Supplementary file 1 [file Data_Sheet_1.docx]
